# Sir Astley Cooper on Dislocations and Fractures of the Joints

**Published:** 1822-12-01

**Authors:** 


***A Treatise on Dislocations, and on Fractures of the Joints.*** By Sir Astley Cooper, Bart. F. R. S. Surgeon to the King, &c. One Volume, Quarto, pp. 562, and 30 Plates. Price 1*l.* 11*s.* 6*d.* London, October, 1822.

The short address to the "Students of St. Thomas's and Guy's Hospitals," prefixed to this splendid volume, excited a train of reflections in our minds, which might not be uninteresting, could we afford them space in this article. The address runs thus:—

"My Dear Young Friends,

"This Work was composed for your use, and if you derive advantage from it, my principal object is fulfilled:—but I shall take this opportunity to express my gratitude to you, for the affectionate and respectful manner in which you always receive me as your instructor. Your parents and relatives, many of whom were my pupils, are also entitled to my most grateful acknowledgements:—they fostered me in early life; and by their friendship and recommendation, have largely contributed to procure me a degree of success, I fear, beyond my merits, and a course of uninterrupted happiness which few have been permitted to enjoy.

"Believe me always,

"Your affectionate friend,

"Astley Cooper."

If the life of a medical practitioner is not checkered with great events, his mind is too generally the theatre of anxious solicitude and conflicting emotions. The perplexing difficulties and heavy responsibilities of his avocations, put his reputation daily and hourly in jeopardy—while the rivalry of surrounding competitors is but too well calculated to engender and foster, in the human breast, a host of passions that contribute but little to either health or happiness. He then, who among the favoured few, has left rivalry almost out of sight behind him—whose reputation has become fixed in the minds of his brethren and the public, and, consequently, invulnerable by chance or hostility—whose word is law—and whose very oversights are but foils to his superior judgment, as exceptions prove general rules—he, we say, may be happy, if his own natural disposition will permit. By his cotemporaries, such a man is designated as *fortunate*; though nothing can be more clear, than that he is generally the architect of his own fortunes. True it is, that extensive connexions and ample pecuniary resources, are powerful auxiliaries to talent, and the sine qua non, without it—but very many examples around us demonstrate, that talent, of a certain order, *creates* these for itself, and *commands* them to follow in its train. He is not always the most fortunate man, whose parents are the richest, whose legacies are the largest, or whose friends are the most numerous, when he first starts as a candidate for public favour. We consider it no trifling piece of good luck, in such circumstances, to be born with brains—we do not mean such brains as are daily demonstrated in the dissecting rooms, and which differ little, if at all, from those which we buy for eightpence a pound in a butcher's shop—we mean brains of that kind of texture, that capacitates them for receiving impressions from the external world with accuracy, retaining them with fidelity, combining them with ingenuity, and communicating them with precision. It is only with such a brain, that a man can expect to take the lead in any branch of medical science; and we suspect that it must have been with some such brain that the author of the work before us fought his way, single-handed, to the summit of his profession. It could not have been the attraction of ordinary merit that drew towards its possessor a tide, or rather a torrent of wealth and fame, unprecedented in any age or in any country—and that without the aid of a faction, the patronage of the great, or the inheritance of an ancestral reputation. By a rare union, in fact, of mental energy, physical force, and professional zeal, Sir Astley Cooper has more than realized all that youthful ardour could have anticipated—and that, we firmly believe, without making, or, at least, *deserving*, a single enemy. That such pre-eminence should be attained in the medical, or in any other profession, without exciting *envy*, is as little to be expected as a retrograde motion of the earth in its orbit round the sun; nor is it unamusing to the contemplative philosopher, to observe the *symptoms* of this moral malady, betrayed when most studiously concealed, and revealing its operation where its influence is denied. The following symptom we deem to be nearly pathognomonic of the disease in question. "Sir Astley Cooper is a very good surgeon, *but*, he is not sufficiently *scientific*." Dr. Johnson defines science to be—1st, "*knowledge*"—2dly, "certainty grounded on demonstration." So then, Sir Astley Cooper is a good surgeon—with the trifling exception that he is defective in the *knowledge* of surgery!—On this point we have but two remarks to make. In the first place, were Sir Astley Cooper tried by a jury of his professional brethren in any spot of Europe beyond the sphere of his personal competitors, he would be unanimously acquitted—or, rather, the indictment would be at once thrown out by a grand jury. In the second place, we are confident, that the same verdict would be given, even by a *Middlesex* jury, and that with a most overwhelming majority.*

We have hinted that, if riches, honours, and fame, can make a man happy, our author ought to be happy; and we have his own declaration, in his address, that he really is so. We confess, however, that we are ill-natured enough to hope that this happiness is not complete, and that it will not be so, until this able surgeon shall have communicated to his brethren at large, the fruits of his unparalleled experience. It is fortunate for society, that this last duty of an exalted medical character, is one to which Nature herself most strongly prompts. However long the range of life, and however numerous the wreaths of laurel, few men can be insensible to posthumous fame. The productions of the press are the least perishable of all human memorials. The pyramids could not preserve the memory, or even the names, of their founders; while the Iliad has rendered Homer immortal. Nothing but the press can withstand the scythe of Time—and none but the author of a good book can, with propriety, say, "saltem non omnis moriar." That these "longings after immortality" do not operate equally on all men, is fortunate in some respects, and to be regretted in others. It saves us many a ponderous tome of dulness, but, at the same time, it deprives us of much valuable knowledge which sinks into the grave with its possessor. It unfortunately happens too, that those men, in our profession, who are most capable of imparting valuable information, are least inclined to come before the public as authors. And, indeed, when we reflect that every man considers himself a critic, upon such occasions, and entitled to make all manner of ill-natured observations on a book, we can hardly wonder at the backwardness to authorship among those who are established in reputation without such hazards. It is hardly necessary to remark, that this very consideration enhances our obligations to such men as Sir Astley Cooper, for imparting valuable information to their brethren, at the risk of illiberal criticism for themselves. And this leads us to remark, that the volume before us is just the reverse of books in general—the matter is good, and the price

* With respect to science, our own feelings are, that it does not consist in hypothetical speculations emanating from a feverish brain, and which perish almost in their birth; but in sober and judicious reflexions upon the subjects presented to our senses, and which, if they are fair inductions from careful and accurate observations, will endure for ever. We apprehend that this is the view which our author, also, has taken of science, and his successful and honourable career proves the wisdom and the propriety of his choice.

is low. We are confident that the sale of a large impression will not more than defray the expence of the letter-press and engravings. The latter alone, 30 in number, are fully worth the whole price attached to the volume! The same may be said of Sir Astley Cooper's splendid work on Hernia. The sale of a large edition did not pay for the plates. Such instances of biblical generosity are very unusual in these book-making days, when the most trumpery materials are vamped up and exposed at an enormous price. We do not, indeed, mean to insinuate that other authors can or ought to follow the example of Sir Astley—very few of them are in circumstances to do so:—but still the individual instance of liberality towards his less fortunate brethren, is not the less worthy of commemoration on the part of the author in question.

A considerable part of the volume before us has been previously published in the form of essays, which were reviewed in the second volume of our first, or Quarterly Series, No. 8, for April, 1820; but it was always Sir Astley Cooper's intention to embody the subject of dislocations in a single work. He has, therefore, enlarged and improved the plates, added new matter to that previously published, and has given a view of those dislocations which he had not before described.

> " To those who have purchased my Essays, it would probably be some accommodation, that I should publish the additional matter separately; and if they will send their names to me in less than three months from this publication, expressing such a wish, I will have the subjects not previously described, printed in the octavo form for their convenience." *Preface.*

As our first series is not in the hands of half our present subscribers, and as the size of the volume before us must naturally cramp its circulation among the more distant members of medical society, we shall probably do right to exhibit as full a view of the subject of dislocations generally, as our limits will permit, especially as we confined ourselves in our review of the Essays, to simple and compound dislocations of the ancle-joint *only*.

Sir Astley observes in his preface, that it is probable his professional brethren will be disposed to think he has limited to too short a period, the attempts at reduction of dislocations; yet, having observed that, except in very emaciated, relaxed, and aged persons, the injury done in the extension has been greater than the advantage gained by the reduction, he is not disposed to recommend attempts at the latter, especially in strong muscular persons, after a period of three months from the accident, " finding that the use of the limb is not, when reduced, greater than that which it would have acquired by having remained in its dislocated state." Our author also states, in his preface, an exception to a general rule, which he has lately seen, where the foot was inverted instead of everted in a fracture of the neck of the thigh bone.

In some general and highly judicious observations on dislocations, our author most truly remarks, that students too often neglect to make themselves sufficiently acquainted with the anatomy of the joints, while they take great pains to dissect the muscles of a limb with neatness and minuteness, throwing away the member without any examination of the ligaments, a knowledge of which is of more importance than of the muscles. Sir A. has known even an hospital surgeon apply pullies in case of a fracture of the neck of the thigh bone, which had been mistaken for dislocation, thus exposing the patient to violent and protracted extension. At the same time, it is but justice to acknowledge that the tumefaction arising from extravasation of blood, and the tension resulting from inflammation, render it exceedingly difficult, in the early days of an accident, to be perfectly assured of the exact extent of the injury.

> "And, therefore, conclusions drawn at a time when the muscles are wasted, the swelling dispersed, when the head of the bone can be distinctly felt, and the motions of the limb are found to be impeded in a particular direction, if they tend to the prejudice of the individual who may have given a different opinion under circumstances so much less favourable for forming a just opinion, they will be both illiberal and unjust." 4.

We hope our young surgical brethren will take example from the foregoing remark, and make it a rule of conduct never to reflect on the practice or opinions of their cotemporaries before their patients. The breach of this precept is the bane of our profession—and we are convinced that the *want of liberality* towards each other has injured us ten times more in the eyes of the public than all the instances of ignorance, negligence, and other kinds of misconduct put together. Occasions may occur where we are compelled to expose the errors or the ignorance of our brethren, but we apprehend that they are very rare—at least we have not met with half a dozen cases in our lives where there was an absolute necessity of making the extra-professional party acquainted with the matter.

The immediate effects of dislocation are change of form in the limb, which may be shorter or longer than natural, loss of motion, and pain either obtuse or acute. In most instances the head of the bone can be felt in the new locality, and rotation of the limb reveals the accident. A remote effect is crepitus, produced by the effusion of fibrin into the joint and bursæ, in consequence of which the synovia becomes inspissated, and crackles under motion. Some degree of inflammation and tumefaction succeeds, rendering the detection of the injury much more difficult, and sometimes almost impossible. Occasionally, though rarely, suppuration takes place in the dislocated joint, and the patient is destroyed. If the limb be not reduced, the bone forms for itself, in time, a new bed, and some degree of motion is gradually recovered, especially in the shoulder. In the lower extremity the patient is lame for life in such cases. On dissection, we find the head of the bone displaced; the capsular and other peculiar ligaments torn; (excepting the tendon of the biceps, in shoulder dislocations, which Sir A. has not found torn;) the formation of new capsular ligaments; the articulating head of the bone unchanged, if thrown on a cushion of muscle, but much changed if thrown on bone, or on a thin muscle covering a bone. In the *first* case, (cushion of muscle,) the articulating cartilage remains, a new capsular ligament being formed around but not adhering to it—in the *latter* case, (cushion of bone,) absorption of the periosteum of one bone and cartilaginous surface of the other takes place, thus forming a smooth hollow surface, with an ossific deposit around it, something like the brim of the original socket, demonstrating the amazing powers of nature.

Dislocations occasionally take place from mere relaxation of the ligaments, of which some curious cases are related by our able author. This relaxed state of the ligaments is sometimes produced by an accumulation of synovia in the joints. A case of dislocation of the patella from this cause is detailed by Sir Astley, where the patient could not walk without a tight bandage to keep the knee-pan from slipping. Even the loss of muscular power will sometimes cause dislocation; two instances of which are stated. In one case the loss of muscular tone was produced by over-distention of the muscles—in the other, it was from paralysis of one side, occurring during dentition. Dislocations frequently take place from ulceration, by which the ligaments are detached, and the bones become destroyed. This is often the case in the hip-joint. There is a preparation at St. Thomas's Hospital, where the knee had been dislocated by ulceration, the joint anchylosed, and the leg turned directly forwards at right angles with the femur.

Dislocations are occasionally accompanied by fracture. In such cases it is proper to reduce the dislocation, without loss of time, taking care that the fractured part be strongly bandaged in splints, to prevent any injury to the muscles.

If this be not done at first, it cannot be done afterwards, without risking the disunion of the fracture. Thus, if there be a compound fracture of the leg cotemporaneous with a dislocation of the shoulder, the reduction of the latter should be immediately undertaken as soon as the fractured limb is secured in splints.

With the exception of the first and second vertebræ of the neck, which are said to be occasionally dislocated, what are called dislocations of the spine are really fractures of the vertebræ followed by displacement of the bones, but not of the intervertebral substance.

Compound dislocations—that is, dislocations with exposure of the cavity of the joint from laceration of the integuments and ligaments, are attended with great danger, and on the following account.

"When a joint is opened, inflammation of the lacerated ligaments and synovial membrane speedily succeeds; in a few hours suppuration begins, and granulations arise from the surface of the synovial membrane, which being a mucous membrane, is more disposed to the suppurative, than to the adhesive inflammation. But the same process does not immediately take place upon the extremity of the bone, because it is covered by the articular cartilage. This cartilage, before the cavity fills with granulations, becomes absorbed, by an ulcerative process instituted on the end of the bones, beginning from the synovial membrane. The bone inflames, the cartilage becomes ulcerated; numerous abscesses are formed, in different parts of the joint, and at length granulations spring from the extremities of the bones deprived of their cartilages, and fill up the cavity; generally these granulations become ossified, and anchylosis succeeds; but sometimes they remain of a softer texture, and some degree of motion in the joint is gradually regained." 18.

This process requires great constitutional efforts, and if the constitution be weak, amputation will often be necessary to preserve life. The treatment will be amply discussed under the head of compound dislocation of the ancle joint. Of partial dislocations we need not speak, nor of the causes of dislocations. Of the astonishing strength of muscles, ligaments, and tendons, a horrible and revolting example was furnished at the execution of Damien, for the attempted murder of Louis XV. Four horses were attached to the four extremities of this wretched man, and after dragging fifty minutes in vain, the executioners were obliged to cut the muscles and ligaments with their knives to effect the dismemberment! Happily for the feelings of mankind, such barbarous spectacles are now for ever banished. The privation of life, by the most simple and expeditious means, is sufficiently cruel, humiliating, and denaturalizing; and we hope the day is not far off, (indeed it cannot be,) when the life of man shall, *on no account,* be destroyed by the sword of justice. Hard labour or solitary confinement appears to be the proper expiation of guilt or crime; and even when that crime is murder, the same act, though under another name, should not be committed in open day as the punishment.

Dislocations are comparatively rare in the aged and in children—the bones generally giving way first. What are called dislocations of the hip-joint in children, are considered by Sir Astley as arising from ulceration. Dislocation (supposed) of the elbow-joint, in the same subjects, "is an oblique fracture of the condyles of the os humeri, which produces the appearance of dislocation by allowing the radius and ulna, or the ulna alone, to be drawn back with the fractured condyle, so as to produce considerable projection at the posterior part of the joint."

Our experienced author has clearly shown that it is principally from the muscles we experience difficulty in reducing dislocations—and that from their inherent tonicity rather than their voluntary or involuntary contractions. This kind of contraction is not succeeded by fatigue or relaxation, but will continue an indefinite time, even till the muscle suffers change of structure, increasing daily in its power of resistance from the first occurrence of the accident. It is this resistance from muscles, aided by their voluntary contraction, which it is the business of the surgeon to counteract—and it is easily counteracted if extension be made immediately after a dislocation has happened; but in a few days great difficulty occurs.

The means of reduction are both constitutional and mechanical. It is wrong to use the latter alone, lest the degree in which it is necessary to employ it occasion violence and injury—indeed the most powerful mechanical means will often fail if unaided by constitutional remedies. Bleeding, the warm bath, and nausea, are the principal constitutional aids. Of these, venesection is the most powerful, especially when the blood is drawn from a large orifice, the patient being kept in the erect position. Whether the warm bath be used by itself, or as an auxiliary to the bleeding, it should be from 100 to 110 of temperature, the patient being kept in the bath till the fainting effect is produced, when he should be immediately placed in a chair wrapped in a blanket, and the mechanical means employed. Of late years our author has employed nauseating doses of tartar emetic, principally with the view of keeping up the faintish state produced by the other two means.

The mechanical process is next to be exerted, by fixing one bone (that in which the socket is) and drawing the other towards the socket. The force should be gradually applied, so as to produce that state of fatigue and relaxation which is sure to follow continued extension. The great object is to firmly fix the socket bone—thus, if *one* person pulls at the scapula and *two* at the fore-arm, the scapula is necessarily drawn with the os humeri, and the extension is very imperfectly made. The compound pully is far preferable to the force of assistants. Its effects may be directed by the judgment of the practitioner, whereas the exertions of assistants are sudden, violent, and often ill directed—their action being as likely to lacerate the parts as to restore the bone to its place. In dislocations of the hip-joint pullies should always be employed—and in those dislocations of the shoulder that have remained long unreduced. Sir Astley Cooper thinks it better, as a general rule, to apply the extention to the bone dislocated than to the limb of which that bone forms a part. To this there is an exception in the case of the shoulder; in dislocations of which he places his heel in the axilla and draws at the wrist, in a line with the side of the body, by which means the pectoral muscle and latissimus dorsi are brought into a state of relaxation; whereas, they would form a powerful opposition, if the arm was carried far from the side.

Bandages are generally necessary after reduction, to prevent another dislocation, especially at the shoulder and in the lower jaw. The hip is rarely re-dislocated.

Sir Astley Cooper believes that much mischief is produced by attempts to reduce dislocations of long standing in very muscular persons. He has seen the patient's condition rendered much worse than before by abortive attemps of this kind. Our author is of opinion that three months for the shoulder and eight weeks for the hip may be fixed on as the latest periods for reductive attempts, except in people of extremely relaxed fibre, or of a very advanced age.

Having premised these observations on dislocations in general, we now come to particular instances, beginning with:—

*Dislocations of the Hip-Joint.* The anatomy of this joint is first described by Sir Astley, and then he proceeds to the modes of dislocation. These, in his experience, have been four—viz. upwards on the dorsum ilii—downwards into the foramen ovale—backwards and upwards into the ischiatic notch—and forwards and upwards upon the body of the pubes. No dislocation downwards and backwards, as described by some surgeons, has occurred at Guy's or St.

Thomas's Hospitals within the last thirty years, nor in our author's private practice. Sir A. therefore doubts, but does not deny its existence.

The dislocation upwards on the dorsum ilii is the most frequent of occurrence, and is known by the following signs:—

"The limb on the dislocated side is from one inch and half to two inches and half shorter than the other, as is well seen by comparing the malleoli interni, and by bending the foot at right angles with the leg. The toe rests against the tarsus of the other foot; the knee and foot are turned inwards, and the knee is a little advanced upon the other. When the leg is attempted to be separated from the other it cannot be accomplished, for the limb is firmly fixed in its new situation, so far as regards its motion outwards; but the thigh can be slightly bent across the other. If the bone be not concealed by extravasation of blood; the head of the thigh-bone can be perceived during rotation of the knee inwards, moving upon the dorsum of the ilium, and the trochanter major advances towards its anterior and superior spinous process, so as to be felt much nearer to it than usual. The trochanter is less prominent than that on the opposite side, for the neck of the bone and the trochanter are resting in the line of the surface of the dorsum ilii; upon a comparison of the two hips, the roundness of the dislocated side will be found to have disappeared. A surgeon, then, called to a severe and recent injury of the hip-joint, looks for a difference in length, change of position inwards, diminution of motion, and decreased projection of the trochanter." 40.

The accident most likely to be confounded with the dislocation upwards, is fracture of the neck of the thigh bone within the capsular ligament. Sir Astley observes, that the marks of distinction are generally sufficiently strong to prevent an error in a person commonly attentive. We shall give the distinctions in our excellent author's own words.

"In a fracture of the neck of the thigh-bone, the knee and foot are generally turned outwards; the trochanter is drawn upwards and backwards, resting upon the dorsum ilii: the thigh can be readily bent towards the abdomen, although with some pain: but, above all, the limb which is shortened from one to two inches, by the contraction of the muscles, can be made of the length of the other by a slight extension, and when the extension is abandoned, the leg is again shortened. If, when extended, the limb is rotated, a crepitus can often be felt, which ceases to be perceived when rotation is performed under a shortened state of the limb. Fracture of the neck of the thigh-bone within the capsular ligament, rarely occurs but in advanced age, and it is the effect of the most trifling accident, owing to the interstitial absorption which this part of the bone undergoes at advanced periods of life. Fractures externally to the capsular ligament occur at any age, but generally in the middle periods of life; and these are easily distinguished by the crepitus which attends them, if the limb be rotated, and the trochanter compressed with the hand. The position is the same as in fractures within the ligament. Fractures of the neck of the thigh-bone are very frequent accidents when compared to dislocations." 41.

Our author's first plate exhibits these dislocations in a visible—we had almost said a tangible form.

To confound dislocations from violence and diseases of the hip-joint, betrays culpable ignorance of anatomy, or reprehensible want of observation. The gradual progress of the symptoms; the pain in the knee; the apparent elongation at first, and real shortening afterwards; the capacity for motion, yet the pain on extreme rotation, flexion, and extension, are marks of difference which ought to strike the most inattentive observer. It is true that the consequences of a disease of this kind, when it has long existed, are, ulceration of the ligaments, acetabulum, and head of the bone, allowing such a change of relative situation of parts, as sometimes gives the limb the position of dislocation—it is the history of the case which easily decides the question.

The cause of this dislocation is generally a fall, when the knee and foot are turned inwards—or a blow, when the limb is in that position. The head of the bone is then displaced upwards, and turned backwards.

" In the reduction of this dislocation, the following plan is to be adopted: take from the patient from twelve to twenty ounces of blood, or even more, if he be a very strong man; and then place him in a warm-bath at the heat of 100°, and gradually increase it to 110°, until he feels faint. During the time he is in the warm-bath, give him a grain of tartarized antimony every ten minutes until he feels some nausea, then remove him from the bath and put him in blankets, and place him between two strong posts about ten feet asunder, in which two staples are fixed; or rings may be screwed into the floor, and the patient be placed upon it. My usual method is to place him on a table covered with a thick blanket, upon his back; then a strong girt is passed between his pudendum and thigh, and this is fixed to one of the staples. A wetted linen toller is to be tightly applied just above the knee, and upon this a leather strap is buckled, having two straps with rings at right angles with the circular part. The knee is to be slightly bent, but not quite to a right angle, and brought across the other thigh a little above the knee of that limb. The pullies are fixed in the other staple, and in the straps above the knee. The patient being thus adjusted, the surgeon slightly draws the string of the pulley, and when he sees that every part of the bandage is upon the stretch, and the patient begins to complain, he waits a little to give the muscles time to fatigue; he then draws again, and when the patient complains much, again rests, until the muscles yield. Thus he gradaally proceeds until he finds the head of the bone approach the acetabulam. When it reaches the lip of that cavity, he gives the pulley to an assistant, and desires him to preserve the same state of extension, and the surgeon then rotates the knee and foot gently, but not with a violence to excite opposition in the muscles, and in this act the bone slips into its place." 43.

The surgeon must not expect to hear the bone snap when it goes in, because the muscles are so much relaxed by the process above-described, that they have not power to act with violence. It is only by loosening the bandages and comparing the limbs that the surgeon becomes assured of the reduction. When there is difficulty in bringing the bone over the lip of the acetabulum, it may be lifted by placing the arm under it near the joint; or a napkin may be placed under it as near the head of the bone as possible, and raised by an assistant.

Several very interesting and highly illustrative cases are introduced by our author from page 45 to page 63, which we must pass over, in order to give as much of our author's valuable didactic matter as possible.

*Dislocation downwards, into the Foramen Ovale.* This generally happens when the thighs are widely separated from each other. The ligamentum teres, and the lower part of the capsular ligament, are torn through, and the head of the bone becomes situated in the posterior and inner part of the thigh, upon the obturator externus muscle.

" The limb is in this case two inches longer than the other. The head of the bone can be felt by pressure of the hand, upon the inner and upper part of the thigh towards the perineum, but only in very thin persons. The trochanter major is less prominent than on the opposite side. The body is bent forwards, owing to the psoas and iliacus internus muscles being put upon the stretch. The knee is considerably advanced if the body be erect; it is widely separated from the other, and cannot be brought without great difficulty near the axis of the body to touch the other knee, owing to the extension of the glutei and pyriformis muscles. The foot, though widely separated from the other, is neither turned outwards nor inwards generally, although I have seen it varying a little in this respect in different instances; but the position of the foot does not in this case mark the accident. The bent position of the body, the separated knees, and the increased length of the limb, are the diagnostic symptoms. The position of the head of the bone is below, and a little anterior to the axis of the acetabulum; and a hollow is perceived below Poupart's ligament." 66.

Sir Astley states the particulars of an interesting dissection which he many years ago made of an accident of this kind, the preparation of which is now in St. Thomas's Hospital.

The head of the thigh bone was found resting in the foramen ovale, around which bony matter was deposited, so as to form a deep cup, in which the head of the thigh bone was inclosed, but in such a manner as to allow of considerable motion. The cup thus formed surrounded the neck of the thigh bone, without touching it, so inclosing its head that it could not be removed from the new socket without breaking its edges. The inner surface of this new cup was extremely polished. The original acetabulum was half filled up with bone. The head of the thigh bone was very little altered—the articulating cartilage still remaining—the ligamentum teres broken—the capsular ligament partially torn through. The preparation exhibits an astonishing instance of the powers of Nature in compensating for injuries.

"The reduction of this dislocation is generally very easily effected. If the accident has happened recently, all that is required is, to place the patient upon his back, to separate the thighs as widely as possible, and to place a girt between the pudendum and upper part of the luxated thigh, fixing it to a staple in the wall. The surgeon then puts his hand upon the ancle of the dislocated side, and draws it over the sound leg, and the head of the bone slips into its socket." 67.

It is proper, and generally necessary, to fix the pelvis, by a girt passed around it and crossed under that which passes around the thigh, otherwise the pelvis moves in the same direction with the head of the bone. A very illustrative plate is given, shewing the mode of reduction in this species of dislocation. Where the dislocation has existed for some weeks, Sir Astley recommends the patient to be placed on his sound side—to fix the pelvis by one bandage, and to carry another under the dislocated thigh to which the pullies are to be affixed perpendicularly—then to draw the thigh upwards, whilst the surgeon presses down the knee and foot, to prevent the lower part of the limb being drawn with the thigh bone. Great care must be taken not to advance the leg in any considerable degree, otherwise the head of the thigh bone will be forced behind the acetabulum into the ischiatic notch, whence it cannot afterwards be removed. Here our author has introduced some interesting cases (not in the original essays) illustrative of this species of accident, which the surgical reader will find it very advantageous to peruse.

*Dislocation backwards into the Ischiatic Notch.* In this accident the head of the femur lodges on the pyriform muscle, between the edge of the bone which forms the upper part of the ischiatic notch, and the sacro-sciatic ligaments, behind the acetabulam, and a little above the level of the middle of that cavity. This is the most difficult dislocation both to distinguish and to reduce.

"The signs of this dislocation are, that the limb is about half an inch to one inch shorter than the other, but generally not more than half an inch; that the trochanter major is behind its usual place, but is still remaining nearly at right angles with the ilium, with a slight inclination towards the acetabulum. The head of the bone is so buried in the ischiatic notch, that it cannot be distinctly felt except in thin persons, and then only by rolling the thigh-bone forwards as far as the comparatively fixed state of the limb will allow. The knee and the foot are turned inwards, but not near so much as in the dislocation upwards, and the toe rests against the ball of the great toe of the other foot. When the patient is standing, the toe touches the ground; but the heel does not quite reach it. The knee is not so much advanced as in the dislocation upwards, but is still brought a little more forwards than the other, and is slightly bent. The limb is fixed, so that flexion and rotation are in a great degree prevented." 76.

It is generally produced by force being applied when the body is bent forwards upon the thigh, or when the thigh is bent at right angles with the abdomen, in which position, if the knee be pressed inwards, the head of the bone is thrown behind the acetabulum.

"The reduction of the dislocation in the ischiatic notch is generally extremely difficult, and is best effected in the following manner: the patient should be laid on a table upon his side, and a girt placed between the pudendum and the inner part of the thigh to fix the pelvis. Then a wetted roller is to be applied around the knee, and the leather strap over it. A napkin is to be carried under the upper part of the thigh. The thigh-bone is then brought across the middle of the other thigh, measuring from the pubis to the knee, and the extension is to be made with the pullies. Whilst this is conducting, an assistant pulls the napkin at the upper part of the thigh with one hand, and rests the other upon the brim of the pelvis, and thus lifts the bone as it is drawn towards the acetabulum over its lip. For the napkin I have seen a round towel very conveniently substituted, and this was carried under the upper part of the thigh, and over the shoulders of an assistant, who then rested both his hands on the pelvis, as he raised his body and lifted the thigh" 78.

Sir Astley has here introduced a new and interesting case, communicated by Mr. Rogers, an intelligent surgeon of Manningtree. The patient, in a drunken frolic, received an injury while wrestling or fighting with one of his companions, but Mr. R. did not see him till the next day, when the whole of the right thigh and of the soft parts around the pelvis were immensely swoln. It was therefore impossible to ascertain the nature of the injury, though Mr. R. suspected some unusual dislocation of the thigh from observing the knee and foot very much turned inwards, the limb being scarcely shortened. Local and general means were used for the reduction of the swelling and inflammation, and the patient was kept quiet for eleven days. Mr. Nunn of Colchester, and Mr. Travis of East Bergholt, now assisted Mr. Rogers with their advice. They all agreed that there was a dislocation, but of what kind they could not ascertain. They resolved to delay a few days, and, in the interim, procure Sir Astley's Essay then recently published. In it they found the case described. By imitating exactly the process recommended by Sir Astley, they happily succeeded in reducing the dislocation. Preparatory to the operation, they bled the patient *ad deliquium*, and while fixing the pullies, gave him four grains of tartar emetic at intervals, to produce nausea. We think it highly probable, that this, and many other men since, owe the free use of their limbs to the admirable Essays in question.

Sir Astley Cooper, in his remarks on this case, observes that the descriptions given of dislocation into the ischiatic notch by authors, are very incorrect—and must have occasioned great errors in practice. Thus, it has been stated that the limb is shorter than the other in such accidents—an error that must have arisen from examining a pelvis separated from the skeleton and there remarking that the ischiatic notch is below the level of the acetabulum when the pelvis is horizontal—although it is really above the acetabulum in the natural oblique position of the bony circle, at least as regards the horizontal axis of the two cavities. It is to be remembered, that there is no such accident as dislocation of the hip downwards and backwards.

*Dislocation on the pubes.* This is the most easily detected of all; and generally happens from a person, while walking, putting his foot into some unexpected hollow in the ground, his body at the moment being bent backwards. The head of the bone is thus thrust forward on the pubes. The distinctive symptomatology we shall give in the language of the author, which is a model of terse and luminous description.

"In this species of dislocation, the limb is an inch shorter than the other; the knee and the foot are turned outwards, and cannot be rotated inwards, but there is a slight flexion forwards and outwards; and in a dislocation which had been long unreduced, the motion of the knee backwards and forwards was full twelve inches; but the striking criterion of this dislocation is, that the head of the thigh-bone may be distinctly felt upon the pubes, above the level of Poupart's ligament, on the outer side of the femoral artery and vein.

It feels as a hard ball there, which is readily perceived to move by bending the thigh-bone." 94.

Easy as this accident is of detection, our author has known three instances, in which it was overlooked till too late. Of one of these the preparation is now in St. Thomas's Hospital —one was a gentleman from the country, in whom the accident was not discovered until some weeks had elapsed, when he submitted to an unsuccessful attempt at reduction—the third was a patient in Guy's Hospital, who was admitted for an ulcerated leg, and was found to have a dislocation upon the pubes that had happened some years previously.

"In the reduction of this dislocation, the patient is to be placed on his side on the table; the girt to be carried between the pudendum and inner part of the thigh, and fixed in a staple, a little before the line of the body. The pullies are fixed above the knee, as in the dislocation upwards, and then the extension is to be made in a line behind the axis of the body, the thigh-bone being drawn backwards. After this extension has been for some time continued, a napkin is to be placed under the upper part of the thigh, and an assistant, pressing with one hand on the pelvis, lifts the head of the bone, by means of the napkin, over the pubes and edge of the acetabulum." 96.

As far as the experience and enquiries of our author enable him to calculate, he thinks that the relative proportion of dislocations at the hip-joint stands nearly as follows:—Of 20 cases, 12 will be on the dorsum ilii—five in the ischiatic notch—two in the foramen ovale—and one on the pubes. It is evident, however, that this calculation is liable to considerable error from the extreme difficulty of making it.

It is not a little curious, that the once celebrated Sharpe, formerly surgeon to Guy's Hospital, and author of a Treatise on Surgery, who had a large share of the public and private practice of the metropolis, did not believe that a dislocation of the thigh-bone ever occurred! Yet, in the present day, our provincial surgeons readily detect such injuries, and generally succeed in reducing them. "Let them never forget, however, that it is to their knowledge of anatomy, more especially of morbid anatomy, that they are indebted for this superiority." Sir Astley quotes the case related by Mr. Cornish, of Falmouth, (published in a former number of this Journal,) where a dislocation of the hip was reduced by a fall on board a vessel, five years after the accident. Sir Astley has searched the books of both St. Thomas's and Guy's Hospitals, without being able to find the name of Mac Fadder; but, possibly, he may have entered (as many sailors do) under an assumed name. Mr. Cornish will doubtless make enquiries about this.

We have now finished the subject of dislocations of the thigh-bone, and it is almost unnecessary to remark, that Sir Astley Cooper has conferred a great obligation on his surgical brethren, for the very important information which he has thus communicated to them, in such clear and explicit language that the veriest tyro can comprehend, with ease, the whole of the diagnostic and therapeutical indications. We now come to—

*Fractures of the Os Innominatum.* As these accidents are liable to be mistaken for dislocations, and as any extension would be productive of serious, or even fatal consequences, our author is anxious to say a few words concerning them in this place.

"When a fracture of the os innominatum happens through the acetabulum, the head of the bone is drawn upwards, and the trochanter somewhat forwards, so that the leg is shortened, and the knee and foot are turned inwards: such a case then may be readily mistaken for dislocation into the ischiatic notch. If the os innominatum is disjointed from the sacrum, and the pubes and ischium are broken, the limb is a slight degree shorter than the other; but in this case the knee and foot are not turned inwards. Of the first of these accidents I have seen two examples; of the latter only one.

"These accidents are generally to be detected by a crepitus being perceived on the motion of the thigh, if the hand be placed upon the crista of the ilium; and they are attended with more motion than occurs in dislocations." 105.

The above precepts are illustrated by three interesting cases, two at St. Thomas's, the other at Guy's Hospital. To these we must refer for particulars. All three patients died, and the dissections are given.

One of the most interesting and valuable portions of the work before us, is that on fractures of the upper part of the thigh-bone, commonly called:—

*Fracture of the Neck of the Femur.* This accident is but too frequently confounded with dislocations of the the thigh-bone, and no wonder, since "it must be confessed, that their discriminating marks are sometimes with difficulty detected." Sir A. avers that three distinct species, very different in their nature, in their treatment, and in their results, have been described and classed under the indiscriminate appellation of "fracture of the neck of the thigh-bone." Hence, he thinks, have arisen the differences of opinion, which have led to great doubt and discussion respecting the process which Nature employs for their cure. He justly observes, that less hypothetical reasoning, and more attention to the development of such accidents, *by dissection,* would have prevented much of the discussions and discrepancies in question.

These accidents are more frequent than dislocations of the thigh-bone. Thus, in St. Thomas's and Guy's Hospitals, not more, on an average, than two dislocations are annually seen; whereas, the wards are seldom without an example of fracture.

The three species of fracture are as follows:—1st. Fracture through the neck of the bone, entirely *within* the capsular ligament. 2dly. Fracture through the neck of the thigh-bone, *at its junction with the trochanter major*, and consequently, *external* to the capsular ligament. 3dly. Fracture through the trochanter major, *beyond its junction with the cervix femoris.*

1. *Within the Capsular Ligament.* The appearances which are produced by this fracture, we shall give in the words of our author, as we cannot abbreviate the language without destroying the sense.

" The leg becomes from one to two inches shorter than the other, for the connexion of the trochanter major with the head of the bone by means of the cervix being destroyed by the fracture, the trochanter is drawn up by the muscles as high as the ligament will permit, and consequently rests upon the edge of the acetabulum and upon the ilium above it. This difference in the length of the limbs is best observed by desiring the patient to place himself in the recumbent posture on his back, when, by comparing the malleoli, it will be found that one leg is from one to two inches shorter than the other. The retraction thus produced is at first easily removed, by drawing down the shortened limb, when it will appear of the same length with the other; but immediately this extension is abandoned, the muscles draw it into its former position; and this appearance may be repeatedly produced by extending the limb. This evidence of the nature of the accident continues until the muscles acquire a fixed contraction, which enables them to resist any extension which is not of the most powerful kind." 118.

Another circumstance which marks the nature of this injury is, the *turning outwards of the foot and knee*—a state produced, in great part at least, by the numerous and strong rotatory muscles of the hip-joint, which proceed from the pelvis to be inserted into the thigh-bone, and to which, very feeble antagonists are provided.

" Directly that the bed clothes are removed, two circumstances strongly arrest the attention of the surgeon, namely, the diminished length of the injured limb, and the eversion of the foot and knee. In the dislocation upwards, the head and neck of the bone prevent the trochanter from being drawn backwards, whilst the broken and shortened neck of the thigh-bone in fracture of this part, readily admits it; and hence the reason why the foot is inverted in the one case, and everted in the other." 119.

A few hours must elapse before the muscles acquire this fixed contraction—which is the reason that this accident has been mistaken for dislocation, and, consequently, that patients, even in hospital practice, have been exposed to useless and painful extensions. In this species of fracture, the patient suffers but little pain when perfectly at rest in the horizontal posture; "but any attempt at rotation is painful," more especially rotation inwards, because the broken extremity of the bone then rubs against the lining of the capsular ligament. The pain which is felt in this accident is in the upper and inner part of the thigh, opposite to the insertion of the iliacus and psoas muscles into the trochanter minor, or, sometimes, just below this point. The perfect extension of the thigh may be easily effected, but flexion is more difficult, and somewhat painful, especially in directing the thigh towards the pubes. In this accident, the trochanter major is drawn upwards towards the ilium, and the broken neck of the bone, attached to the trochanter, is placed nearer the spine of the ilium than the trochanter itself, by which alteration of position, the trochanter projects less on the injured, than on the sound side, and, consequently, is much more concealed than naturally, until the muscles waste from the duration of the injury, when it can be distinctly felt upon the dorsum ilii.

"In order to form a still more decided judgment of this accident, after the patient has been examined in the recumbent posture, let him be directed to stand by his bed-side, supported by an assistant, so as to bear his weight upon the sound limb; the surgeon then observes most distinctly the shortened state of the injured leg, the toes rest on the ground but the heel does not reach it; the knee and foot are everted, and the prominence of the hip is diminished. On ordering the patient to attempt to bear upon the injured limb, he finds himself incapable of doing so, without considerable pain, which seems to be produced by the psoas, iliacus, and obturator externus muscles being put upon the stretch in the attempt, as well as by the pressure of the broken neck of the bone against the interior surface of the capsular ligament, and there will be a greater or less projection of the trochanter, proportional to the length of the fractured cervix femoris attached to it." 121.

*Crepitus* is not discoverable here when the patient is resting on his back with the limb shortened; "but, if the leg be drawn down, so as to bring the limbs to the same length, and rotation be then performed, the crepitus is sometimes observed from the broken ends of the bone being thus brought into contact—but the rotation inwards most easily detects it." 122. When the patient is standing on the sound limb, with the fractured limb unsupported, rotation *inwards* will sometimes discover the crepitus, as the weight of the limb brings the broken bones in apposition. This species of fracture is rarely seen in males, while the wards of an hospital are seldom without instances in females, especially aged females. "The more horizontal position of the neck of the bone, and the comparative feebleness of the female constitution, are probably the causes of this peculiarity." The fracture within the capsule seldom happens but at an advanced period of life. Hence, our author imagines, has arisen the great confusion among surgeons of the highest character, respecting the nature of this fracture—for it has been represented, as happening at a period of life in which it never takes place. Old age, it is true, is an indefinite term; for in some, it is as strongly marked at sixty, as in others, at eighty years. "That regular decay of Nature which is called old age, is attended with changes which are easily detected in the dead body; and one of the principal of these is found in the bones, for they become thin in their shell, and spongy in their texture." 123. Hence, the bones of old persons may be cut with a pen-knife, which is incapable of making any impression on them at the middle periods of life.

"Even the neck of the thigh-bone in old persons, is sometimes undergoing an interstitial absorption, by which it becomes shortened, altered in its angle with the shaft of the bone, and so changed in its form as to give an idea, upon a superficial view, of its having been the subject of fracture, so as to lead persons into the erroneous supposition of the bone having been partially broken and re-united: but it requires very little knowledge of anatomy, to distinguish, in the skeleton, the bone of advanced age from that of the middle period of life." 124.

This fracture, Sir Astley observes, very rarely occurs under fifty years of age; and dislocation as rarely above that period; although there are exceptions, of course, to both these rules. Between fifty and eighty years is the most common period at which the fracture occurs; for, from the different state of the bone, the same violence which would produce dislocation in the adult, occasions fracture in the aged. That this state of bone in old age favours much the production of fractures, is shewn by the slightest cause often occasioning them. In London, the most common cause is slipping from the flags down on the carriage pavement, though it is a descent of only a few inches. Another frequent cause is a *slight* fall on the trochanter major. Our author lays emphasis, and very properly, on the term *slight*, in order that the young surgeon may be on his guard against the supposition, that so important an injury must be the result of excessive violence. "Such an opinion is as liable to be injurious to his reputation, as that of confounding this accident with dislocation."

It has been asserted, that these fractures unite like those of other parts of the body ; but the dissections which our author has made in early life, and the opportunities he has since had of confirming these observations, have convinced him, that the transverse fracture of the cervix femoris within the capsular ligament, does not unite by a bone—a circumstance which he has taught in his lectures for thirty years—"and this is a most essential point, as the reputation of the surgeon hinges upon it." Thus, Sir Astley was called to a case of this fracture, where the medical attendant had been promising, week after week, a union of the fracture, and the restoration of a sound and useful limb. After many weeks, the patient became anxious for further advice, and our author did all in his power to lessen the impression which the mistake had made; but he could not alter the result, which, of course, belied the confident predictions of the surgeon, and injured him in the eyes of the patient at least. It unfortunately happened too, that, in this case, the patient did not recover in the *degree* they usually do. We quote the following passage, and wish it to be engraven on the mind of every student.

"Young medical men find it so much easier a task to speculate than to observe, that they are too apt to be pleased with some sweeping conjecture, which saves them the trouble of observing the processes of nature; and they have afterwards, when they embark in their professional practice, not only every thing still to learn, but also to abandon those false impressions which hypothesis is ever sure to create. Nothing is known in our profession by guess: and I do not believe, that from the first dawn of medical science to the present moment, *a single correct idea has ever emanated from conjecture:* it is right, therefore, that those who are studying their profession should be aware that there is no short road to knowledge; and that observations on the diseased living, examination of the dead, and experiments upon living animals, are the only sources of true knowledge; and that inductions from these are the sole bases of legitimate theory." 127.

We think the expression marked in italics, (by us) is rather too strong; and we could bring forward proofs of the justice of this opinion. We shall only allude to one very curious instance. Galen *conjectured* (for he had no proof whatever) that there was one set of nerves for sensation, and another for motion. If there be any faith in recent experiments on living animals, this conjecture of the ancient physician is likely to prove a truth, and a very curious one too.* The general bearing, however, of the passage, offers most salutary counsel.

Although Sir Astley has never met with a single instance of bony union in transverse fractures of the cervix femoris within the capsular ligament, yet he is not so dogmatical as to assert that such never occurs, especially when we consider the varieties of direction in which a fracture may take place, and the various degrees of violence by which it may have been produced—as, for instance, when the fracture is through the head of the bone, and there is no separation of the fractured ends; or, where the bone is broken without its periosteum and the reflected ligament which covers its neck being torn—or when it is broken obliquely, partly within, and partly external to, the capsular ligament. After all, Sir Astley only wishes it to be understood that, if ever union takes place under the circumstances in question, it must be a very rare occurrence, as he has not yet met with a single example of it. The reasons which Sir Astley assigns for the non-union are as follow:—

*First*—the want of proper apposition in the bones: "for if the broken extremities, in any part of the body, be kept much asunder, ossific union is prevented." This is proved by fractures of the patella, which are known to unite only by ligament—by experiments made on animals, where pieces of the radius, for instance, were cut out, leaving the ulna to prevent apposition, in which case, no ossific union took place—and, lastly, by examples in the human body, some of which we shall quote presently. But first, it is proper to state, that a case is related by Mr. Dunn, in the last volume of the Medico-Chirurgical Transactions, which *seems* to militate against Sir Astley's position. Mr. Dunn's case will be found in our Periscope, to which we refer for particulars. It is only necessary to state here, that three inches of the tibia were removed from a boy's leg, where the ends of the bone projected, and where the fibula was fractured in two places. The leg was only an inch shorter than the other ultimately, though the bones were prevented from coming within an inch and a half, or two inches of each other, by the fibula. All the proofs we have, in this case, of *ossific* union, are grounded on

---

* We are aware that Galen made numerous experiments on animals before he came to this conclusion; but neither he, nor any man yet, has been able to shew the slightest proof, that *separate* nerves were employed for sensation and motion. It is becoming probable that, in the *same* nerve, there is a portion of its structure derived from the cerebrum, and another portion from the cerebellum, and that, to this is owing the diversity of function in, apparently, the same nerve.

the facts, that the boy was able to walk without crutches, and that, by compressing the space between the bones on each side, Mr. Dunn "could trace a continued line of bone." Now we must confess that this evidence of feeling through the integuments appears to us very equivocal—and nothing but post-mortem demonstration would induce us to place implicit belief in the physiological fact or assumption. In respect to the evidence, grounded on the boy's being able to walk, we shall quote the following case from the *Medical Records and Researches*, as related many years ago by Mr. Smith of Bristol.

"The boy was admitted into the Bristol Infirmary for disease of the tibia; and the diseased portion, not exceeding more than from two to three inches in length, that part of the bone was removed by the saw. In a month the limb had acquired so much firmness, that the boy was permitted to walk about the ward, which he was able to perform tolerably well, and in six weeks no doubt was entertained of ossification having taken place in the uniting substance; at this time he sickened with small-pox and died.——Upon examination, the edges of the extremities of the tibia were found absorbed and rounded; and on the inferior portion, a bony callus had formed, about three quarters of an inch in extent; no ossific matter was discoverable in the greater part of the space originally occupied by the diseased bone, but a tough though thin ligamentous band extended from the superior to the inferior portion of the tibia."

In the above case it appears, that had the boy not died, it would have been firmly believed that ossific union had taken place. The fractures in the fibula, also, throw some doubt on the real state of the physiological question, as far as Mr. Dunn's case is concerned. In fine, from what we have seen, both in animals, and in human bones, that had been prevented from approximating, we are disposed to think that Mr. Dunn is deceived—and that the broken tibia is become agglutinated ossifically and ligamentously to the *fibula*, which, in such circumstances, becomes greatly enlarged, and thus adds to the stability and firmness of the limb, while the intermediate space between the ends of the *tibia* becomes filled up by a firm, ligamentous substance, that has given Mr. Dunn the idea of a bony continuity.

The case in question, however, does not, in the least degree, affect the position of Sir Astley, respecting non-union of the neck of the thigh-bone within the capsular ligament—which non-union depends on another principle, as the reasons which we are in the course of developing will evince.

*Second Reason:*—The want of pressure of one bone upon the other, even where the length of the limb is preserved—a circumstance that will operate in preventing ossific union in cases where the capsular ligament is not torn, and it has not been found torn in any of those cases examined by our author. The cause of this want of pressure is thus explained by Sir Astley :—

"From the increased determination of blood to the capsular ligament and synovial membrane, a superabundance of serous synovia, that is, synovia much less mucilaginous than usual, distends the ligament, and thus entirely prevents the contact of the bones, by pushing the upper end of the body of the thigh-bone from the acetabulum. After a time, this fluid becomes absorbed, but not until the inflammatory process has ceased, and ligamentous matter has been effused into the joint, from the interior of the synovial membrane." 130.

That pressure between the broken extremities of bones conduces greatly to their union, is well shewn where two broken bones overlap each other. On that side on which they are pressed together, there will be found an abundant ossific deposit, but scarcely any change on the opposite sides.

"When a fracture occurs amidst muscles, those which are inserted into the fractured part of the bone have generally a tendency to keep the extremities of the bones together, with some few exceptions; but when a fracture occurs in the neck of the thigh-bone, the muscles have only an influence upon one portion of the fractured bone; and this influence serves to draw one part from the other." 131.

*Thirdly*. But the third and principal reason assigned by our author for the want of union in this fracture is the absence of ossific action in the head of the thigh-bone, when separated from its cervix, its life being then solely supported by the ligamentum teres, which has only a few minute vessels ramifying from it to the head of the thigh-bone—circumstances very beautifully shewn in the plate connected with this part of the subject.

"But here it may be observed, that the neck and head of the thigh-bone are naturally supplied with blood by the periosteum of the cervix, and by the reflected membrane which covers it; and that when the bone is fractured, if the periosteum be torn through, and the reflected membrane be broken, to which there can be only very rare exceptions, all the means of ossific action are, in consequence of such fracture and laceration, necessarily destroyed in the head of the bone. Scarcely any change therefore takes place in the head or neck of the bone; no deposit of cartilage or bone similar to that of other fractured bones, is produced; but the deposit which does take place, as will be seen in the plate of fracture of the neck of the thigh-bone, is a deposition of ligamentous matter, covering the surface of the cancellated structure." 132.

*Appearances Post-mortem.* The head of the bone remains in the acetabulum attached by the ligamentum teres. Upon parts of the bone are small white specks covered by the articular cartilage. The cervix is sometimes broken directly transverse, at others with obliquity. The cancellated structure of the broken surface of the head and cervix is hollowed by the occasional pressure of the neck attached to the trochanter and consequent absorption—and this surface is sometimes partially coated with ligamentous deposit. The cancelli are rendered firm and smooth by friction. Portions of the head of the bone are broken off, and either floating loosely, or attached by means of ligament. In respect to the neck attached to the trochanter, it is, in a great degree, absorbed, and but a small portion of it remains. Its surface is yellow and resembling ivory, if the bones have rubbed together. In some examples of this fracture Sir Astley has seen a few ossific deposits manifested around this small remaining part of the neck of the bone, and also upon the trochanter major and thigh bone below it. The capsular ligament becomes much thickened, and the synovial lining greatly changed by inflammation, being also much thickened, and containing a large quantity of serous synovia, mixed with flakes of ligamentous matter formerly produced by inflammation of the membrane.

It is well known that two specimens of (supposed) union by bone of the cervix femoris have been sent to the College Museum. That sent by Mr. Liston of Edinburgh is the most curious and most imposing; but on minutely examining it, a few months ago, we became convinced that there never was an entire fracture of the cervix within the capsular ligament. We observe also, that the late Mr. Wilson expressed himself thus:—"I have examined very attentively these two preparations, and cannot perceive one decisive proof, in either, of the bones having been actually fractured."

"It appears then, from this account of the dissection of those whose bodies are examined after having suffered from this fracture, that no ossific union is produced; that nature makes slight attempts for its production upon the neck of the bone, and upon the trochanter major; but scarcely any upon the head of the bone; and that if any union be produced, it is by ligament only." 135.

Our author made several experiments on living animals with the view of illustrating this point; but, owing to the difficulty of effecting a fracture of the cervix femoris in the proper place, he only succeeded in four instances. These all confirmed the deductions already before our readers. They may be seen in the collection at St. Thomas's Hospital.

*Treatment.* Various modes of management have been proposed and adopted in the treatment of this fracture; but in no one instance, as far as our author is acquainted, with success. We shall therefore be excused from detailing the mechanical measures which have been employed, and merely state what Sir Astley himself is inclined to adopt.

"Baffled in our various attempts at curing these cases, and finding the patient's health suffering under the trials made to unite them, I should, if I sustained this accident in my own person, direct, that a pillow should be placed under the limb throughout its length, that another should be rolled up under the knee, and that the limb be thus extended for ten days or a fortnight, until the inflammation and pain have subsided. I should then daily rise and sit up in a high chair, in order to prevent a degree of flexion which would be painful. Our hospital patients, treated after this manner, are allowed in a few days to walk with crutches; after a time, a stick is substituted for the crutches, and in a few months they are able to use the limb without any adventitious support.

"The degree of recovery, in these cases, is as follows: if the patient be very corpulent, the aid of crutches will be for a long time required; if less bulky, a stick only will be sufficient; and where the weight of the body is inconsiderable, the person is able to walk without either of these aids, but drops a little at each step on that side, unless a shoe be worn having a sole of equal thickness to the diminished length of the limb. In every case, however, in which there is the smallest doubt whether it be a fracture within, or external to the ligament, it will be proper to treat the case as if it were the fracture which I shall hereafter describe, and which admits of ossific union." 144.

As danger to life is sometimes involved in these accidents, especially in old and infirm persons, the surgeon should be guarded in his opinion as to the result. "Lameness, says our experienced author, in the transverse fracture, is sure to follow; but its degree cannot, at first, be exactly estimated." Finally, we may state that the dissections of that excellent anatomist and surgeon, Mr. Colles of Dublin, confirm the doctrines delivered by Sir Astley Cooper for thirty years past in the Borough School—a coincidence which must be gratifying to the distinguished author of the work under review.

*Fracture of the Cervix external to the Capsule.* The symptoms of this accident in some respects so nearly resemble those of the fracture within the capsule, that considerable attention is required to distinguish them accurately—for their results are very different. We cannot refrain from introducing the diagnostic symptomatology in the plain and expressive language of the author.

"In this accident the injured leg is but little shorter than the other; the foot and toe on that side are everted, from the loss of support which the body of the thigh-bone sustains in consequence of the fracture; much pain is felt at the hip, and on the inner and upper part of the thigh, and the joint loses its usual roundness. These, then, are all marks of similarity between the two accidents; but still there are many distinguishing signs. First; This accident occurs frequently at the earlier periods of life; for it happens in the young, and in the adult under fifty years of age: I have known it at a later period, but less frequently; therefore, when the above symptoms are seen at any age under fifty years, it will be generally found to be a fracture external to the capsular ligament, and capable of having ossific union produced in it.

"Secondly; These cases may be also in some measure distinguished by the severity of the accident which produces them; whilst the internal fracture happens from very slight causes, this, on the contrary, is produced either by severe blows, from falls from a considerable height, or from laden carriages passing over the pelvis.

Thirdly; It may be generally known by the crepitus which usually attends it upon slight motion, for it is rarely necessary to draw the limb down, to distinguish the grating of one bone upon the other, and this arises from the less retraction of the limb." 146.

This accident is much more painful than fracture within the ligament, especially on motion—the leg and thigh become much swollen—there is high irritative fever—and many months elapse before the patient recovers the use of the limb; which is rarely more than an inch shorter than the other.

On dissection, the seat of the fracture is found to vary much, in different examples; but it is always external to the capsular ligament—in general it is at the root of the cervix femoris. Here our author introduces valuable cases from Mr. Powell of Surrey Street, Mr. Wray of Fleet Street, Mr. Travers and Mr. Oldnow of Nottingham, for which we must refer to the original.

"We now see the reason of the difference of opinion respecting the union of the fracture of the neck of the thigh-bone. In the internal, the bones are not applied to each other, and the nutrition of the head of the bone is imperfect, but in the external, the bones are held together by the surrounding parts, and easily kept in apposition by external pressure." 155.

In the treatment of this injury the length of the limb is preserved by applying a roller around the foot of the injured leg, and by binding the foot and ancles firmly together, so as to prevent their retraction, and thus render the uninjured side the splint to that which is fractured, giving it a continued support. A broad leather strap should also be buckled round the pelvis, including the trochanter major, to press the fractured portions of the bone firmly together. The following plan our author has also known to succeed:—

"The patient being placed on a mattrass on his back, the thigh is to be brought over a double inclined plane composed of three boards, one below, which is to reach from the tuberosity of the ischium to the patient's heel, and the two others, having a joint in the middle by which the knee may be raised or depressed; a few holes should be made in the board admitting a peg which prevents any change in the elevation of the limb, but that which the surgeon directs; over these a pillow is thrown to place the patient in as easy a position as possible." 156.

When the limb has been thus extended, a long splint is to be placed upon the outer side of the thigh, to reach above the trochanter major, and to the upper part of this is fixed a strong leather strap, which buckles round the pelvis, so as to press one portion of the bone upon the other. The lower part of the splint is to be fixed with a strap round the knee, to prevent its position being moved. The limb must be kept very steady for two months, at the end of which time the patient may be permitted to rise from his bed, provided the attempt does not give him much pain; but he is still to retain his outer splint for a fortnight longer, with the straps round the pelvis. By this treatment he ultimately recovers a very good use of his limb.

*Fracture of the great Trochanter.* This sometimes happens through the trochanter major obliquely, the cervix femoris not participating. It occurs at every period of life, and the symptoms, according to Sir Astley, are these:—the leg is very little, and sometimes not at all, shorter than the other—the foot is benumbed—in some cases the patient is unable to turn in bed without assistance, and the attempt gives him great pain. The broken portion of the trochanter major is, in some cases, drawn forwards toward the ilium; in others, it falls towards the tuberosity of the ischium; but it is generally widely separated from that portion which remains connected with the neck of the bone. The foot is greatly everted—the patient cannot sit, without much pain—crepitus is with difficulty discovered if the trochanter is either much fallen, or much drawn forwards.

"The distinguishing marks of this accident are, eversion of the foot and the altered position of the trochanter major, attended with crepitus under very extended motion of the upper part of the limb; and a little diminution of the length of the limb." 158.

This fracture Sir Astley has found to unite very firmly, and more quickly than when the cervix is broken at the root of the trochanter. The patient recovers with a very good use of his limb.

A very interesting case of this kind is drawn up by Mr. Harris, of Reading, in the management of which Sir Astley Cooper and Mr. Brodie assisted. Two or three other very illustrative cases are introduced by the able author himself, for the details of which we must refer to the original work.

*Fractures below the Trochanter.* When this fracture is just below the two trochanters, it is a difficult accident to manage, and great distortion is the consequence of mismanagement. The iliacus internus and psoas muscles, assisted perhaps by some others, draw the broken bone forwards and upwards, so as to form nearly a right angle with the body. If pressure be made on the projecting bone in this case, it only adds to the patient's sufferings, without preserving the bone in its proper site. This union exceedingly overlaps, and is very feeble.

"To prevent this horrid distortion and imperfect union, two principles are required to be strictly observed; the one is to elevate the knee very much over the double inclined plane, and the other to place the patient in a sitting position, well supporting him by pillows during the process of union; the degree of elevation of the body which is required, will be readily ascertained by observing the approximation of the fractured extremities of the bones; and this position is demanded, to relax the psoas and iliacus muscles, and thus prevent the elevation of the upper part of the bone. In this manner, and by this only, can the great deformity I have described be prevented. When by this posture the extremities of the bones are brought into proper apposition, and all projection of its upper portion is removed, either the splints may be applied which are commonly used in fracture of the thigh-bone, or, what is better, a strong leather belt lined with some soft material, should by means of several straps be buckled around the limb." 175.

We have thus almost insensibly extended the review of a single division of the work before us to a long article; and must therefore reserve the rest for our next number. The subject is highly important, and we are anxious that the pages of our Journal should contain a greater than usual proportion of materials from a work that bears the impress of one of the first masters of surgery in the present advanced state of that science. Sir Astley Cooper has produced a GREAT BOOK—and he has put it out of the power of the severest cynic to say that, in this case, it is a GREAT EVIL. On the contrary, we very much fear that we shall not "meet its like again" in our day—unless from the author himself. We sincerely hope that Sir Astley Cooper will continue (we were going foolishly enough to say at his *leisure* moments) to commit to the imperishable records of the press a portion, at least, of that immense store of valuable and practical knowledge of which he is the depository. By this process he will give a kind of ubiquity to himself, and thus confer a lasting obligation on his profession, and, through it, on mankind at large.

